# Synthesis, Characterization and Application of Amine-Functionalized Hierarchically Micro-Mesoporous Silicon Composites for CO_2_ Capture in Flue Gas

**DOI:** 10.3390/molecules27113429

**Published:** 2022-05-26

**Authors:** Yilan Chen, Junjie Wu, Xin Wang, Minyi Liu, Yamin Liu

**Affiliations:** 1 School of Ecological Environment and Urban Construction, Fujian University of Technology, Fuzhou 350118, China; chenyilan1982@163.com (Y.C.); 15613613422@126.com (J.W.); wxzd321@163.com (X.W.); 19841522@fjut.edu.cn (M.L.); 2 Fuzhou Smart Environmental Industry Technology Innovation Center, Fuzhou 350118, China

**Keywords:** CO_2_ adsorbent, hierarchically micro-mesoporous silicon, amine-functionalized, kinetic

## Abstract

An efficient CO_2_ adsorbent with a hierarchically micro-mesoporous structure and a large number of amine groups was fabricated by a two-step synthesis technique. Its structural properties, surface groups, thermal stability and CO_2_ adsorption performance were fully investigated. The analysis results show that the prepared CO_2_ adsorbent has a specific hierarchically micro-mesoporous structure and highly uniformly dispersed amine groups that are favorable for the adsorption of CO_2_. At the same time, the CO_2_ adsorption capacity of the prepared adsorbent can reach a maximum of 3.32 mmol-CO_2_/g-adsorbent in the actual flue gas temperature range of 303–343 K. In addition, the kinetic analysis results indicate that both the adsorption process and the desorption process have rapid adsorption/desorption rates. Finally, the fitting of the CO_2_ adsorption/desorption experimental data by Avrami’s fractional kinetic model shows that the CO_2_ adsorption rate is mainly controlled by the intra-particle diffusion rate, and the temperature has little effect on the adsorption rate.

## 1. Introduction

For climate change, the ultimate global goal is to avoid dangerous disturbances to the climate system. To achieve this goal, UNFCCC member states have shown strong support for measurable guardrail targets, such as “Avoid 1.5 °C” or “Avoid 450 ppm CO_2_”. However, the CO_2_ concentration in the atmosphere has increased sharply from 340 ppm in 1980 to 408 ppm in 2019 [1], and if existing human activities do not sufficiently and quickly change, the global temperature increase will exceed 1.5 °C. Therefore, a variety of methods should be adopted to reduce CO_2_ levels, such as planting more trees, replacing fossil energy with renewable energy, improving the efficiency of coal-fired plants, and using CO_2_ capture and storage (CCS) to reduce CO_2_ content [2,3].

CCS technology is an attractive method because it helps reduce the release of CO_2_ into the environment and allows the continued use of coal to meet the world’s energy needs [2,4]. According to different coal-fired plant configurations, three main approaches can be applied to reduce CO_2_ emissions from the flue gas of these plants, including pre-combustion capture, oxyfuel combustion, and post-combustion capture [5]. Among these CCS technologies, post-combustion capture technology (which uses wet/dry adsorbents to absorb CO_2_ from the flue gas that is produced by the combustion of fossil fuels) is the most popular industrial method [6]. Post-combustion capture technologies generally include absorption [7], porous materials adsorption [8], membrane separation [9], cryogenics and hydration etc. [5]. At present, some experiments and computational studies have been conducted on the above-mentioned technologies [10,11,12].

Among these technologies, the adsorption-based technology is the most attractive because it has the potential to lower costs while avoiding the defects of aqueous amine absorption [13]. Adsorbents are the foundation for the adsorption process. In recent years, many porous solid adsorbents have been investigated in order to capture CO_2_ from flue gas, including carbon materials [14,15,16], zeolites [17], mesoporous silicon [18], pillared lamellar clays [19], and the metal-organic frameworks (MOFs) [20,21].

The ideal solid adsorbent for CO_2_ capture should have good CO_2_ selectivity, a high CO_2_ adsorption capacity, and a faster CO_2_ adsorption/desorption rate [22]. The faster the CO_2_ adsorption/desorption process the more economical the process of CCS [23]. Usually, the microporous materials, such as the MCM series and ZSM series, have a large CO_2_ adsorption capacity because of their high surface area and high porosity [24]. However, due to their rapid decrease in adsorption capacity at higher temperatures, these microporous materials are mainly used at low temperatures (below 303 K) [25]. Mesoporous materials such as SBA series and KIT series have unique mass-transfer properties due to their large pore diameters [26]. It is therefore expected that a new type of micro-mesoporous composite material with the advantages of high surface area and rapid mass transfer can be prepared through the combination of microporous and mesoporous materials production. In addition, this material may display a better CO_2_ adsorption performance after surface amine functionalization; this is because amines are nucleophilic, and they can strongly interact with electrophilic CO_2_ through nucleophilic substitution reactions to increase the CO_2_ adsorption capacity.

Therefore, the objective of this study is to synthesize an amine-functionalized hierarchically micro-mesoporous adsorbent and discuss its CO_2_ adsorption behavior. In order to achieve this target, firstly, the nano-scale microporous silicon precursor was assembled into an ordered cubic mesoporous structure by a two-step hydrothermal crystallization method to synthesize the micro-mesoporous silicon material. Then, the prepared micro-mesoporous silicon material was used as a support, an amino compound was used as a modifier, and the amine-functionalized hierarchically micro-mesoporous silicon composites was fabricated by an impregnation method. After that, the pore structure, surface characteristics and CO_2_ adsorption performance of the prepared adsorbent were analyzed. Finally, the experimental data of isothermal CO_2_ adsorption/desorption was simulated by a mathematical model, and the kinetic characteristics of CO_2_ adsorption/desorption were discussed.

## 2. Materials and Methods

### 2.1. Materials

Tetrapropylammonium hydroxide solution (TPAOH); aluminium isopropoxide; tetraethylorthosilicate (TEOS); polyethylene-polypropylene glycol (P123); hydrochloric acid (HCl); and n-Butanol were used for the synthesis of a hierarchically micro-mesoporous silicon.

Ethylenediamine (EDA); diethylenetriamine (DETA); tetraethylenepentamine (TEPA), pentaethylenehexamine (PEHA); ethylene imine polymer (PEI); and ethanol were used for the synthesis of an amine-functionalized hierarchically micro-mesoporous adsorbent. The chemicals used in the experiment are listed in Table 1. All chemicals were used without further purification.

### 2.2. Sample Preparation

#### 2.2.1. Synthesis of Support Material

The preparation of the hierarchically micro-mesoporous silicon material was as follows: First, 4.54 g of TPAOH was dissolved in 16.87 g of deionized water at room temperature and stirred for 30 min, and then 0.17 g of aluminum isopropoxide and 0.1 g of NaOH were added. After stirring for 30 min, the mixture was heated to 303 K, and 8.5 g of TEOS was dropped into the mixture. After 16 h of vigorous stirring, the final solid–liquid mixture was transferred to a Teflon kettle and subjected to hydrothermal treatment at 423 K for 24 h. The microcrystalline emulsion was then cooled to room temperature and the final resultant was labeled as microcrystalline emulsion A.

Second, 4.0 g of P123 (5800) was dissolved in a mixture of 144 g of H_2_O and 7.9 g of hydrochloric acid solution (35 wt.%) at 313 K under stirring. After the P123 was completely dissolved, 4.0 g of n-Butanol was added. After stirring continuously for 1 h, the above microcrystalline emulsion A was added and sonicated for 30 min. After vigorously stirring at 313 K for 24 h the resultant was transferred to a Teflon kettle and subjected to hydrothermal treatment at 373 K for 24 h. The solid–liquid mixture was filtered to obtain a solid product and the solid product was dried at 373 K for 10 h. After that, the obtained solid sample was calcined at 823 K for 6 h under a temperature programmed system (5 K/min) to remove the template. The resulting material is herein denoted as micro-mesoporous silicon (MMS).

#### 2.2.2. Preparation of Amine-Functionalized Hierarchically Micro-Mesoporous Adsorbent

The amine-loaded hierarchically micro-mesoporous adsorbent was produced by a wet impregnation method. The ethylenediamine (EDA); diethylenetriamine (DETA); tetraethylenepentamine (TEPA); pentaethylenehexamine (PEHA); and polyetherimide (PEI) were used as amine-modified materials. In a typical experiment, 1 g of amine-modified material was dissolved in 50 mL of ethanol at room temperature, and after stirring for 30 min, 1 g of MMS was added, and then the mixture was stirred for 2 h. After that, the mixture was evaporated at 353 K and then dried in air at 373 K for 1 h. Finally, a white composite was obtained. The obtained composites are denoted as AMMS-E, AMMS-D, AMMS-T, AMMS-P and AMMS-PEI, respectively. Here, the AMMS represents amine-functionalized micro-mesoporous silicon, and the E, D, T, P, and PEI stand for EDA, DETA, TEPA, PEHA, and PEI, respectively.

### 2.3. Characterization

The surface crystallinity of the AMMS was analyzed by the X-ray diffraction (XRD) measurement using a Rigaku powder diffractometer (D8 ADVANCE, Bruker, Bremen, Germany) with Cu Kα radiation (λ = 0.15406 nm). The tube voltage was 45 kV and the current was 40 mA. The XRD diffraction patterns were taken in the 2*θ* range of 0.5–10° and 5–60° at a scan speed of 2°/min. The surface morphology was determined with a scanning electron microscope (S4800, Hitachi, Tokyo, Japan).

The surface area analyzer (Autosorb iQ, The Quantachrome Instruments U.S., Boynton Beach, FL, USA) was used for the nitrogen adsorption/desorption test. Before the measurement, the sample was degassed at 573 K under nitrogen flow for 3 h. The surface area of the powder was obtained using the Brunauer-Emmett-Teller (BET) method. The pore size distributions were calculated by the Barrett-Joyner-Halenda (BJH) equation. The total pore volume was determined from the amount of adsorbed N_2_ at *P/P*_0_ = 0.99.

A TG (TG 209F3, Netzsch, Selb, Germany) instrument was used to analyze the thermal stability of AMMS, and the thermogravimetric analysis test was performed at a temperature of 303 K to 973 K with a heating rate of 10 K/min under a dynamic N_2_ atmosphere.

The surface chemical groups were analyzed by Fourier Transform Infrared (FTIR, Nicolet IS50, Thermo Fisher Scientific, Waltham, MA, USA) spectroscopies.

### 2.4. Adsorption/Desorption of CO_2_

A TGA device (TG209F3, Netzsch) was used for the CO_2_ adsorption/desorption test. A total of 10 mg of AMMS samples were loaded into a 0.05 cm^3^ platinum sample pan. After stabilization, each sample was heated to 423 K in a nitrogen stream (120 cm^3^ min^−1^), then cooled to the adsorption temperature, and a CO_2_ (99.99%)/N_2_ (99.99%) mixture (10 Vol.% CO_2_) was introduced until the mass of the sample no longer increased (about 50 min). The adsorption capacity was calculated based on the AMMS sample mass increase using Equation (1).

(1) $$q_{a}=\frac{M_{1}-M_{0}}{44\times M_{0}}\times 10^{3}$$

where *q_a_* is the adsorption capacity of CO_2_, mmol g^−1^; *M*_0_ is the mass of pure adsorbent, and g; *M*_1_ is the mass of the sample after adsorbing CO_2_ g.

Once the adsorption process reached equilibrium, the temperature programmed desorption (TPD) test was performed on the same experimental system. The adsorbent was heated to the required desorption temperature (383 K) in a nitrogen stream (120 cm^3^ min^−1^). The total amount of CO_2_ was calculated based on the loss of sample mass using Equation (1).

### 2.5. Adsorption/Desorption Kinetic Models

Avrami’s fractional kinetic model (Equation (2)) is used to study the kinetics of CO_2_ adsorption on AMMS materials. Usually, Equation (2) can be transformed into Equation (3) in the form of desorption component *y*. Thus, Equation (3) can be employed to further understand the desorption kinetics of the CO_2_ desorption process [27].

(2) $$q_{t}=q_{e}\left[1-e^{-{\left(k_{a}t\right)}^{n}}\right]$$

(3) $$y=1-e^{-{\left(k_{a}t\right)}^{n}}$$

where $q_{e}$ and $q_{t}$ (mmol·g^−1^) denote the equilibrium capacity and the adsorption capacity at any time *t* (s), respectively, $k_{a}$ is the kinetic constant of the Avrami model, the n represents as the Avrami exponent, which is often in fractional form, and reflects possible mechanism changes in the adsorption process [28].

## 3. Results and Discussions

### 3.1. Characterization of the AMMS

#### 3.1.1. XRD Analysis

**Figure 1:**
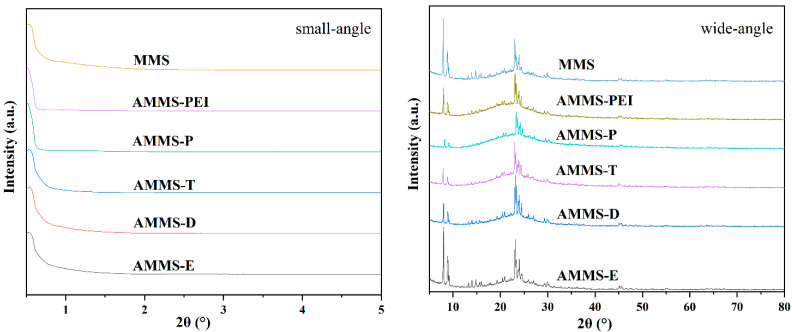
XRD patterns of MMS and AMMS.

Figure 1 provides the small-angle XRD pattern of the MMS and AMMS powder. The spectrum for the MMS and AMMS powder reveals a strong peak at about 2θ = 0.55° that corresponds to the (100) crystal face, indicating that the composites materials MMS and AMMS may have a p6 mm hexagonal-symmetry mesoporous structure that is similar to SBA-15 [29]. However, we cannot find the (110) and (200) peaks that are normally observed in SBA-15 from the spectra of the MMS and AMMS powders, which may indicate that the mesoporous system of MMS and AMMS has become disordered [30]. This disorder may be attributed to the formation of microporous structures that are similar to ZSM-5 materials in MMS and AMMS materials. Figure 1 also displays the XRD patterns in the wide-angle of the MMS and AMMS powder. The sharp peaks that are clearly visible at 2θ = 7.9°, 8.8°, 20.3°, 23.1° and 23.9° correspond to (101), (200), (103), (501), and (303) crystal plains and consistent with the patterns of ZSM-5 that are reported in the literature [31].

The locations of the characteristic Bragg diffraction peaks of the MMS and AMMS samples are almost the same, but the peak intensity (XRD patterns in wide-angle) of the AMMS is reduced, indicating that the amine-modified material is loaded into the micro-pores of MMS. At the same time, the micro-mesoporous structure of MMS is retained.

#### 3.1.2. SEM Analysis

**Figure 2:**
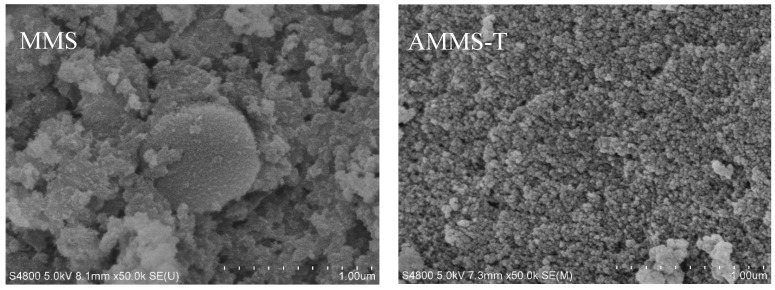
SEM images of MMS and AMMS-T.

The surface morphology of MMS and AMMS-T is presented in Figure 2, and the SEM images of other AMMS are shown in Figure S1. The SEM micrograph of MMS shows that this solid is formed by the aggregation of small amorphous material units (like the ZSM-5 material [31], and a certain number of pores are formed between the units. In contrast to the surface morphology of the MMS material, the SEM micrograph of AMMS-T shows that the surface of AMMS-T is distributed with highly dispersed and uniform particles. This result indicates that the amine-modified material was successfully impregnated into the pores of the MMS material, and the loading of the amine-modified material resulted in a decrease in the surface area and pore volume of the structure, which was further confirmed in the BET surface area analysis. At the same time, the highly uniformly dispersed amine on the solid surface may be beneficial to the adsorption of CO_2_ [32]. The subsequent CO_2_ adsorption analysis also proved this possibility.

#### 3.1.3. BET Analysis

**Figure 3:**
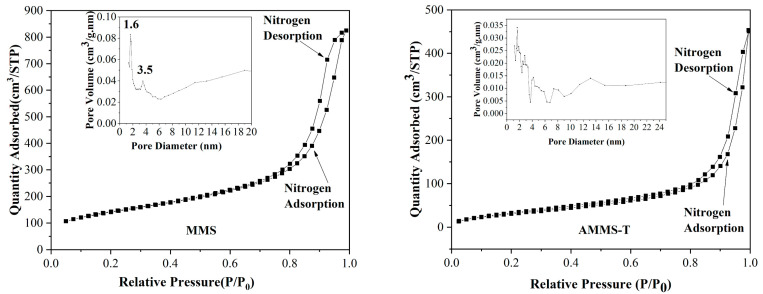
The nitrogen adsorption isotherms of MMS and AMMS-T.

Through nitrogen adsorption experiments at 77 K, the structural characteristics of MMS and AMMS, such as surface area, average pore size and pore volume were analyzed. The nitrogen-adsorption isotherms of MMS and AMMS-T are presented in Figure 3, and the nitrogen adsorption isotherms of other AMMS are displayed in Figure S2. All adsorption isotherms represent the typical type IV features of mesoporous silica. The pore size distribution of MMS and AMMS-T are also displayed in Figure 3. The pore size distribution curve of MMS has two obvious peaks at 1.6 nm and 3.5 nm, indicating that its pore structure is mainly composed of micro-pores and meso-pores. However, the pore size distribution of AMMS-T is relatively random and irregular. This phenomenon may be caused by the disordered accumulation of amine-modified materials on the surface of the pores during the impregnation process.

Table 2 summarizes the surface area, average pore size and pore volume of all the materials. It was observed that after amine modification, the surface area and pore volume of AMMS were significantly reduced. This can be explained by amine impregnation, where amine material accumulates on the surface of the MMS porous channels, resulting in a decrease in surface area and pore volume. These results are consistent with the XRD measurement results.

#### 3.1.4. TG Analysis

**Figure 4:**
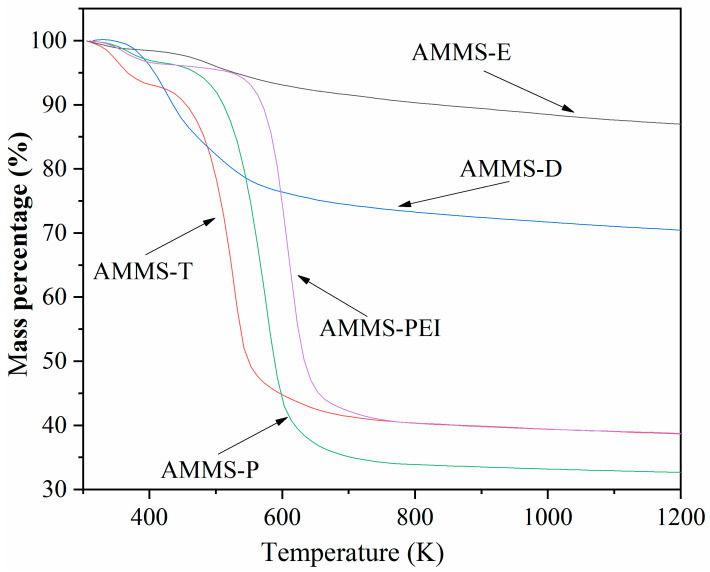
TGA results of AMMS.

The thermal behavior of AMMS was studied by TG analysis. Figure 4 shows the mass loss curve of AMMS. Two stages of mass loss are observed in the TG curve of AMMS. Due to the removal of adsorbed water and CO_2_, the mass loss of 3–8 wt.% in the first stage is approximately between 303 K and 373 K. We noticed that when the temperature is higher than 373 K, the mass loss curves display a relatively straight line within a certain temperature range, indicating that there is basically no mass loss in the sample within this temperature range. For AMMS-T, the temperature range is 373 K to 423 K, AMMS-P is 373 K to 483 K, and AMMS-PEI is 373 K to 543 K. As the temperature continues to rise, the samples begin to undergo the second stage of the mass loss process. The results suggest that AMMS-T, AMMS-E, AMMS-D, AMMS-P and AMMS-PEI have a thermal stability of 423 K, 483 K, 403 K, 483 K, and 543 K, respectively. Finally, when the sample mass remains constant, the total mass loss of AMMS-T, AMMS-P and AMMS-PEI is about 55%, 61% and 55%, respectively. The reason for the different total mass loss of the samples may be attributed to the volatilization and decomposition of different amine-modified materials.

#### 3.1.5. FTIR Analysis

**Figure 5:**
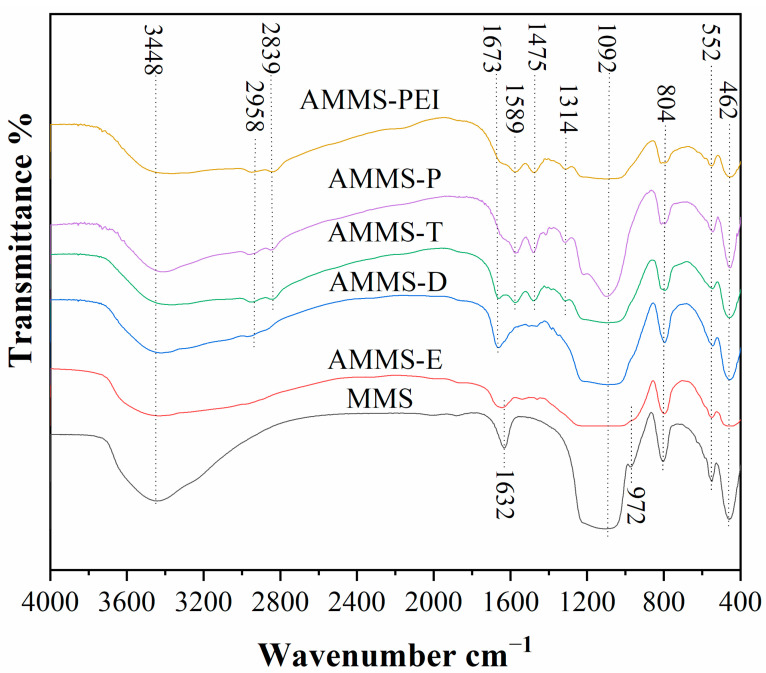
FTIR spectrums of MMS and AMMS.

The FTIR spectrums of MMS and AMMS in the domain of 400–4000 cm^−1^ are demonstrated in Figure 5. For MMS, the adsorption peak around 462 cm^−1^, 804 cm^−1^ and 1092 cm^−1^ could be attributed to the Si-O-Si bending vibrations [33], the symmetric stretching vibrations of Si–O–Si bonds [33], and the asymmetric stretching vibrations of Si–O–Si bonds [34]. Moreover, the adsorption band at 552 cm^−1^ represents the typical vibration band of five- or six-membered rings of X–O–X, where X can be Al or Si [35]. In addition, the adsorption band at 972 cm^−1^ is ascribed to the defective Si–OH group [36]. Furthermore, the adsorption bands at 1632 cm^−1^ and 3448 cm^−1^ are due to the H–O–H bending vibration and the typical of O–H stretching vibration, respectively [37].

The FTIR spectrum of AMMS displays some new adsorption peaks at 1314 cm^−1^, 1475 cm^−1^, 1589 cm^−1^, 1673 cm^−1^, 2839 cm^−1^, and 2958 cm^−1^. The peak at 1314 cm^−1^ is attributed to C–N tensile vibration. The adsorption peaks at 1475 cm^−1^ and 1589 cm^−1^ represent N–H stretching vibrations which are associated with asymmetric and symmetric bending of the primary amines (–NH_2_). In addition, the adsorption peak at 1673 cm^−1^ is related to the bending of secondary amines (–N(R)H) in amine-modified materials. Moreover, the adsorption peaks at 2839 cm^−1^ and 2958 cm^−1^ are due to the CH_2_ asymmetric and symmetric stretching modes of the amine chain in the AMMS. Compared with the MMS [38], we cannot observe the peak at 1632 cm^−1^ from the FTIR spectrum of AMMS. At the same time, the intensity of the peak at 3448 cm^−1^ decreases with the impregnation of amine. The reason for this may be due to the interaction of amine and MMS. Overall, the above FTIR analysis indicates that the amine-modified material is effectively loaded into the pores of the MMS.

### 3.2. Adsorption Properties Analysis

**Figure 6:**
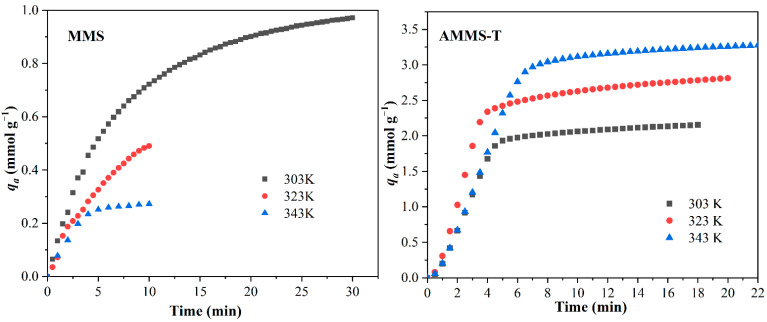
CO_2_ Adsorption curves of MMS and AMMS-T at 303 K, 323 K, and 343 K.

CO_2_ adsorption experiments were carried out at the required temperature with *C*_0_ = 10 vol.% of CO_2_ (inlet CO_2_ concentration). The CO_2_ adsorption capacities (*q_a_*) of MMS and AMMS are presented in Table 3. In addition, the adsorption curves of MMS and AMMS-T at three temperatures (303 K, 323 K, and 343 K) are shown in Figure 6, and the adsorption curves of the other AMMS are displayed in Figure S3.

It can be seen from Table 3 that the *q_a_* of MMS (at different temperatures) is very limited. At the same time, as the temperature increases from 303 K to 343 K, the *q_a_* decreases significantly. This may be due to the exothermic behavior of the adsorption phenomenon. However, the *q_a_* of AMMS-T increases with increasing temperature. The reason for this may be that the loaded amine-modified material has higher molecular activity and mobility at higher temperatures, so the *q_a_* of AMMS shows an increasing trend when the temperature increases from 303 K to 343 K.

From Table 3, we observed that under similar conditions AMMS-T has the best adsorption capacity (*q_a_* = 3.32 mmol-CO_2_/g-adsorbent at 343 K) among all the AMMS. This result is consistent with the results of the SEM analysis. The previous SEM analysis results indicated that among all the surface morphologies of AMMS samples, the surface particle dispersion of AMMS-T was the most consistent and uniform. It is precisely because of the highly uniform dispersion of the amine-modified material particle that agglomeration of the particles is avoided, so that more amine groups of the amine-modified material can contact and react with CO_2_ molecules, thereby improving the ability to adsorb CO_2_.

The adsorption capacity of AMMS-T was compared with the values that were obtained under similar conditions for the CO_2_ adsorbents proposed in the literature (Table 4). We noticed that AMMS-T exhibited a relatively good adsorption capacity under similar conditions.

The abscissa in Figure 6 denotes the time and the ordinate represents the *q_a_*. The overall adsorption curve profiles for 303 K, 323 K, and 343 K display similar adsorptive behavior. All the adsorption processes are completed in a short time, and the adsorption rate is approximately the same. This means that although a higher temperature can increase the CO_2_ adsorption capacity, the adsorption rate is not controlled by the adsorption temperature. The adsorption rate is further analyzed in the subsequent kinetic analysis of the adsorption process.

### 3.3. Adsorption/Desorption Kinetics Analysis

Previous adsorption capacity studies have shown that AMMS-T exhibits the best adsorption capacity among all AMMS (*q_a_* = 3.32 mmol-CO_2_/g-adsorbent, at 343 K). Therefore, AMMS-T was used as a CO_2_ adsorbent to analyze the characteristics of the adsorption/desorption kinetics of the CO_2_ adsorption/desorption process in this study. Multiple adsorption kinetics models have been employed to quantitatively analyze the adsorption and to explore the adsorption mechanism; for example, Lagergren’s pseudo-first-order model [48]; Ho’s pseudo-second-order model [49]; the classical intracrystalline diffusion model [50]; and Avrami’s fractional-order kinetic model [51]. Among these models, since the fractional order of the Avrami model can be used to characterize the complexity of the reaction mechanism or the simultaneous occurrence of multiple reaction paths [52,53], the Avrami fractional-order kinetic model is well suited to analyzing the CO_2_ adsorption kinetics and adsorption mechanisms [54]. For the Avrami model, if *n* = 1, it means that the adsorption process has uniform adsorption [55,56]. If *n* = 2, then the adsorption process may show a perfect one-dimensional growth at the adsorption point after uniform adsorption [57].

**Figure 7:**
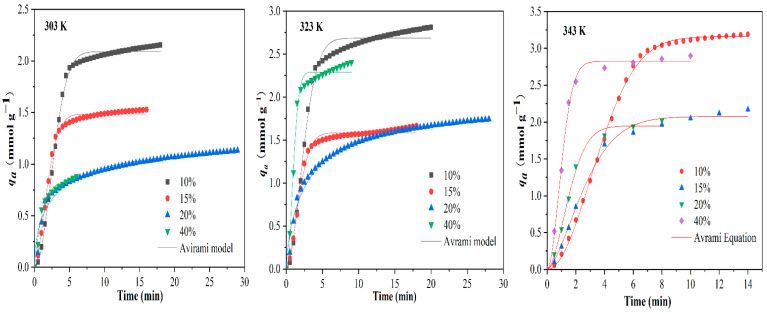
The results of the Avrami kinetics models and the experimental CO_2_ uptakes of AMMS-T.

The kinetics character of CO_2_ adsorption on AMMS-T were investigated by isothermal adsorption at several *T* (303, 323 and 343 K) and at various *C*_0_ (10, 15, 20, and 40 vol.%). The experimental data were simulated using the Avrami model and the results can be found in Figure 7 and Table 5.

As described in Table 5, the adsorption rate constant *k_a_* increases with the increase in *C*_0_ at the same *T*. However, the value of *k_a_* only slightly changes with the increase in *T* under the same *C*_0_. The high partial pressure of CO_2_ can promote the faster diffusion of CO_2_ to the active sites on the surface of the solid pores. Therefore, it can be deduced from the simulation results that *k_a_* is mainly controlled by the intra-particle diffusion process, and temperature has little effect on the *k_a_*.

It can be seen from Table 5 that most Avrami exponent *n* are greater than 1 and less than 2, indicating that additional adsorption may be preferentially provided on the existing adsorption sites and resulting in one-dimensional growth at these adsorption points after uniform adsorption. The value of *n* is less than 2, because as the adsorption progresses, not all the adsorption sites that have adsorbed CO_2_ start a new uniform one-dimensional adsorption, but only some sites continue to adsorb CO_2_; the rest do not continue to absorb CO_2_.

**Figure 8:**
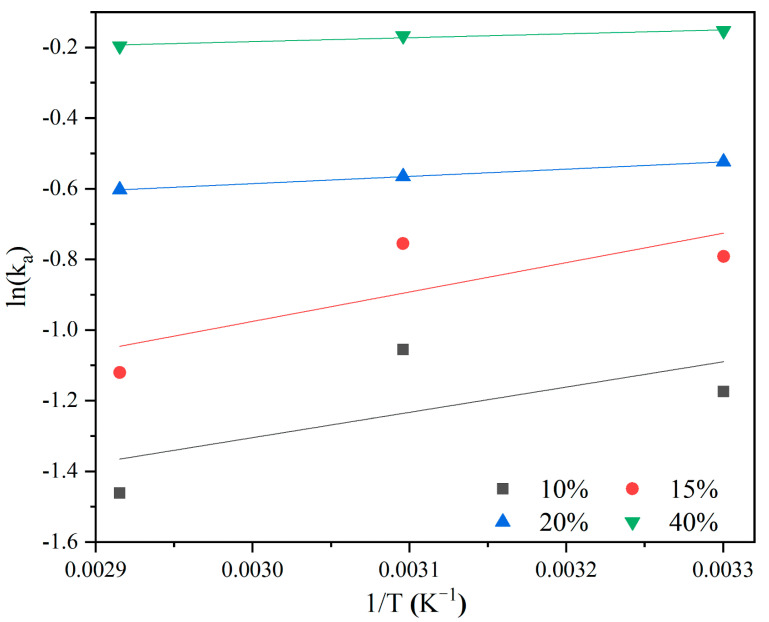
Arrhenius plots for the kinetic constant *k_a_* obtained for the Avrami model.

The *k_a_* can be demonstrated by the Arrhenius equation:

(4) $$k_{a}=Ae^{-\left(E_{a}/RT\right)}$$

where *A* denotes the Arrhenius pre-exponential factor, *E_a_* is the activation energy, and *R* is the universal ideal gas constant. A plot of ln*k_a_* versus 1/*T* is shown in Figure 8. Table 6 presents the *A* and *E_a_* that were obtained by linear regression of the experimental data.

As shown in Table 6, the absolute value of *E_a_* decreases with the increase in *C*_0_, indicating that a high CO_2_ partial pressure promotes the adsorption of CO_2_. The result is consistent with the previous analysis results, that is, the CO_2_ adsorption rate is mainly controlled by the intra-particle diffusion process. The *E_a_* of AMMS-T is less than that of liquid ammonia absorption (40−50 kJ·mol^−1^ [58,59]), which indicates that AMMS-T may be a promising and effective CO_2_ adsorbent.

**Figure 9:**
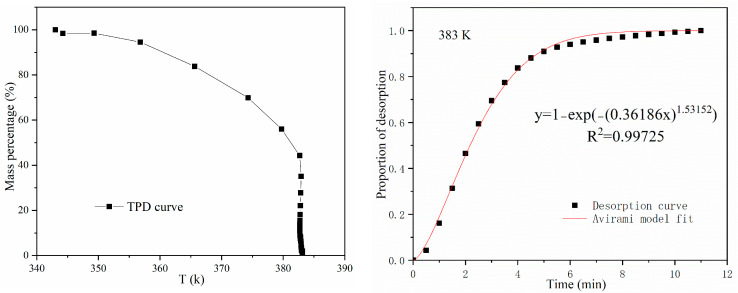
Results of TPD and desorption experiment at 383 K.

Figure 9 displays the TPD curve, the desorption curve at 383 K and the fitting curve of the Avrami fractional kinetic model. The TPD curve is basically a horizontal straight-line segment between 343 K and 353 K, indicating that the adsorbent that was saturated with CO_2_ had almost no mass loss, which means that when T ≤ 353 K, no desorption process occurs. Then, the TPD curve is a downwardly sloping line segment between 353 K and 383 K, and mass loss begins to appear, indicating that CO_2_ is gradually desorbed from the surface of the adsorbent. Finally, when the temperature is maintained at 383 K, the TPD curve presents a vertical line segment, indicating that CO_2_ is quickly and completely desorbed from the surface of the adsorbent. Moreover, according to the desorption curve, the CO_2_ is almost completely desorbed within 6 min, which means that the desorption rate is very fast. All these results suggest that the optimal desorption temperature for AMMS-T is about 383 K.

From Figure 9, we also noticed that the *R*^2^ values for the fitted Avrami fractional-order model are 0.99725, which verifies that the Avrami model can describe the experimental data well.

### 3.4. Cyclic CO_2_ Adsorption/Desorption Behavior of the AMMS-T

**Figure 10:**
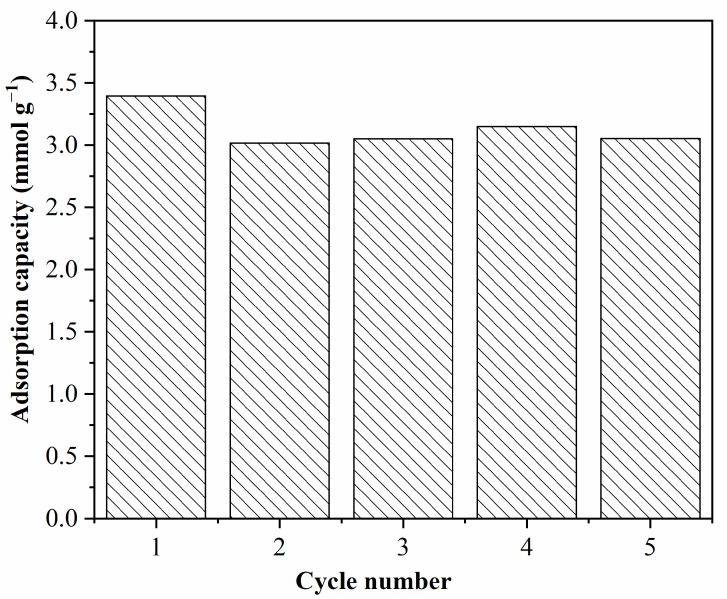
Recycle adsorption/desorption runs of AMMS-T (adsorption/desorption at 343/383 K).

The persistent cyclic adsorption/desorption behavior of the adsorbent is essential for long-term operation. Figure 10 describes the adsorption capacity of the AMMS-T in repeated cycles of CO_2_ adsorption at 343 K and desorption at 383 K. The cycle data indicate that the adsorption performance of the AMMS-T is quite stable, and the adsorption capacity is still about 3.05 mmol g^−1^ after five adsorption/regeneration cycles.

## 4. Conclusions

This study presents an effective amine-functionalized micro-mesoporous silicon adsorbent for CO_2_ capture. The adsorbent was prepared by the impregnation method using micro-mesoporous silicon as a support and TEPA as a modifier. The resulting sample was characterized with the XRD, SEM, TG, nitrogen adsorption and FTIR analysis. The characterization results show that the prepared AMMS-T maintains a micro-mesoporous structure after amine loading, and there are highly uniformly dispersed amine groups distributed on the surface of the pore which is beneficial to the adsorption of CO_2_.

The CO_2_ adsorption capacity of AMMS-T displays an upward trend with the increase in temperature in the range of 303–343 K, and the maximum adsorption capacity is 3.32 mmol-CO_2_ g^−1^-adsorbent. The results of the adsorption kinetic analysis illustrate that Avrami’s fractional kinetic model can fit the CO_2_ adsorption experimental data well. In addition, the simulation results reveal that the CO_2_ adsorption rate of AMMS-T is mainly affected by the intra-particle diffusion rate, and temperature has little effect on it. The optimal regeneration temperature of AMMS-T is about 383 K and the CO_2_ desorption process can be well simulated by the Avrami model.

In summary, the prepared AMMS-T shows excellent CO_2_ adsorption/desorption performance. The good agreement between the results of the Avrami model and the experimental data shows that the kinetic constants that were obtained by the simulation are highly effective for designing the actual CO_2_ adsorption process.

## Figures and Tables

**Table 1:** Chemicals used in the experiment.

Chemical Name	mol. wt.	Purities	CAS-No.	Sources
Tetrapropylammonium hydroxide solution	203.36	25.0%	4499-86-9	Macklin Biochemical Co., Ltd. (Shanghai, China)
Aluminium isopropoxide	204.25	≥98.0%	555-31-7	BASF Biotechnology Co., Ltd. (Hangzhou, China)
Tetraethylorthosilicate	208.33	98.0%	78-10-4	Aladdin Biochemical Technology Co., Ltd. (Shanghai, China)
Polyethylene-polypropylene glycol	~5800		9003-11-6	Macklin Biochemical Co., Ltd. (Shanghai, China)
Hydrochloric acid	36.46	35.0%	7647-01-0	Fuzhou Yihua Chemical Co., Ltd. (Fuzhou, China)
n-Butanol	74.12	≥99.5%	71-36-3	Sinopharm Chemical Reagent Co., Ltd. (Shanghai, China)
Ethylenediamine	60.1	≥99.0%	107-15-3	Sinopharm Chemical Reagent Co., Ltd. (Shanghai, China)
Diethylenetriamine	103.17	99.0%	111-40-0	Aladdin Biochemical Technology Co., Ltd. (Shanghai, China)
Tetraethylenepentamine	189.30	98.0%	112-57-2	Aladdin Biochemical Technology Co., Ltd. (Shanghai, China)
Pentaethylenehexamine	232.38	98.0%	4067-16-7	Macklin Biochemical Co., Ltd. (Shanghai, China)
Ethylene imine polymer	600	99.0%	9002-98-6	Aladdin Biochemical Technology Co., Ltd. (Shanghai, China)
Ethanol	46.07	≥99.7%	64-17-5	Sinopharm Chemical Reagent Co., Ltd. (Shanghai, China)
Carbon dioxide	44.0	≥99.999%	124-38-9	Fuzhou Yuanhua Chemical Co., Ltd. (Fuzhou, China)
Nitrogen	28.0	99.999%	7727-37-9	Fuzhou Yuanhua Chemical Co., Ltd. (Fuzhou, China)

**Table 2:** Nitrogen adsorption/desorption characterization details for MMS and AMMS.

Sample	Surface Area (m^2^·g^−1^)	Total Pore Volume (cm^3^·g^−1^)	Micropore Volume	Mesopore Volume	Average Pore Diameter (nm)
MMS	498	1.261	0.170	1.091	3.5
AMMS-E	404	1.445	0.080	1.365	3.5
AMMS-D	314	1.351	0.140	1.211	1.2
AMMS-T	121	0.770	0.080	0.690	1.6
AMMS-P	88	0.500	0.050	0.450	1.4
AMMS-PEI	9.7	0.084	0	0.084	3.3

**Table 3:** The CO_2_ adsorption capacity of MMS and AMMS.

Adsorbent	Amine-Modified Material	*q_a_* (mmol-CO_2_/g-Adsorbent)		
		303 K	323 K	343 K
MMS *		0.99	0.45	0.33
AMMS-E *	EDA	0.79	0.47	0.37
AMMS-D	DETA	0.70	0.67	0.51
AMMS-T	TEPA	2.23	2.90	3.32
AMMS-P	PEHA	1.41	1.95	2.65
AMMS-PEI	PEI	0.74	1.11	1.75

* *C*_0_ = 100 vol.% CO_2._

**Table 4:** Comparison of CO_2_ adsorption capacity of AMMS-T (present study) with the literature.

Support	Amine Type	Temp. K	CO_2_ Partial Pressure (bar)	CO_2_ Adsorption (mmol-CO_2_/g-ads)	Ref.
HMS	PEI	318	1	2.40	[39]
MS-3040 (Microspherical Silica)	PEI	358	0.95	3.26	[40]
SBA-15	TEPA	333	0.15	2.15	[41]
Zn/CoZIF	PEI	298	1	1.82	[42]
SFM-0.83-100-5.2	PEI	348	0.15	2.48	[43]
Silica	PEI	353	0.15	2.86	[44]
Mesoporous PCN-777	PEI	298	0.25	1.41	[45]
MOF	PEI	298	1	2.84	[46]
MCM550 (Mesoporous Monolithic)	PEI	348	0.12	1.89	[47]
AMMS-T	TEPA	343	0.10	3.32	This work

**Table 5:** The approximate values of the model parameters obtained by Avrami’s model and the corresponding correlation coefficient *R*^2^.

T (K)	*C*_0_ (vol.%)	Avrami Model				*q_a_* (mmol/g)
		*K_a_*	*q_e_* (mmol/g)	*n*	R^2^	
303	10	0.309	2.09	2.000	0.996	2.235
303	15	0.453	1.486	1.681	0.995	1.576
303	20	0.592	1.156	0.543	0.974	1.133
303	40	0.858	0.851	1.024	0.982	1.189
323	10	0.348	2.685	1.813	0.988	2.900
323	15	0.470	1.585	1.731	0.991	1.775
323	20	0.568	1.758	0.653	0.989	1.744
323	40	0.846	2.291	1.880	0.988	2.667
343	10	0.232	3.160	1.982	0.999	3.322
343	15	0.326	2.074	1.549	0.995	2.368
343	20	0.547	1.947	1.750	0.996	2.322
343	40	0.822	2.822	1.882	0.995	3.078

**Table 6:** Related parameters calculated from CO_2_ adsorption isotherms fitted to the Arrhenius Equation.

CO_2_ Concentration (vol.%)	*A* (min^−1^)	*Ea* (KJ/mol)	*R* ^2^
10	0.031696	−5.81806	0.43709
15	0.03105	−6.76534	0.63287
20	0.300659	−1.66947	0.99993
40	0.597542	−0.89788	0.94752

## Data Availability

The data that support the findings of this study are available from the corresponding author, [Liu Y.], upon reasonable request.

## Supplementary Materials

The following supporting information can be downloaded at: https://www.mdpi.com/article/10.3390/molecules27113429/s1, Figure S1: SEM images of AMMS-E, AMMS-D, AMMS-PEI and AMMS-P; Figure S2: N_2_ adsorption isotherms of AMMS-E, AMMS-D, AMMS-P and AMMS-PEI; Figure S3: CO_2_ adsorption curves of MMS, AMMS-E, AMMS-D, AMMS-P and AMMS-PEI.
