# Supplementary material for: Synthesis, Characterization and Application of Amine-Functionalized Hierarchically Micro-Mesoporous Silicon Composites for CO2 Capture in Flue Gas
Source: Molecules. 2022 May 26;27(11):3429. doi: 10.3390/molecules27113429 (PMC9182193; doi:10.3390/molecules27113429)
Supplement: Supplementary file 1 [file molecules-27-03429-s001.zip › molecules-1738446-supplementary.pdf]

Synthesis, Characterization and Application of Amine Functionalized Hierarchically Micro-Mesoporous Silicon Composites for CO_2 Capture in Flue Gas

Yilan Chen [1,2], Junjie Wu[1], Xin Wang[1], Minyi Liu[1,2], Yamin Liu[1,2] *

1. School of Ecological Environment and Urban Construction, Fujian University of Technology, Fuzhou 350118, Fujian, China

2. Fuzhou Smart Environmental Industry Technology Innovation Center, Fuzhou 350118, Fujian, China

Supporting Information Contents:

Figure S1. SEM images of AMMS-E, AMMS-D, AMMS-PEI and AMMS-P

Figure S2. N_2 adsorption isotherms of AMMS-E, AMMS-D, AMMS-P and AMMS-PEI

Figure S3. CO_2 adsorption curves of MMS, AMMS-E, AMMS-D, AMMS-P and AMMS-PEI

Figure S1 SEM images of AMMS-E, AMMS-D, AMMS-PEI and AMMS-P

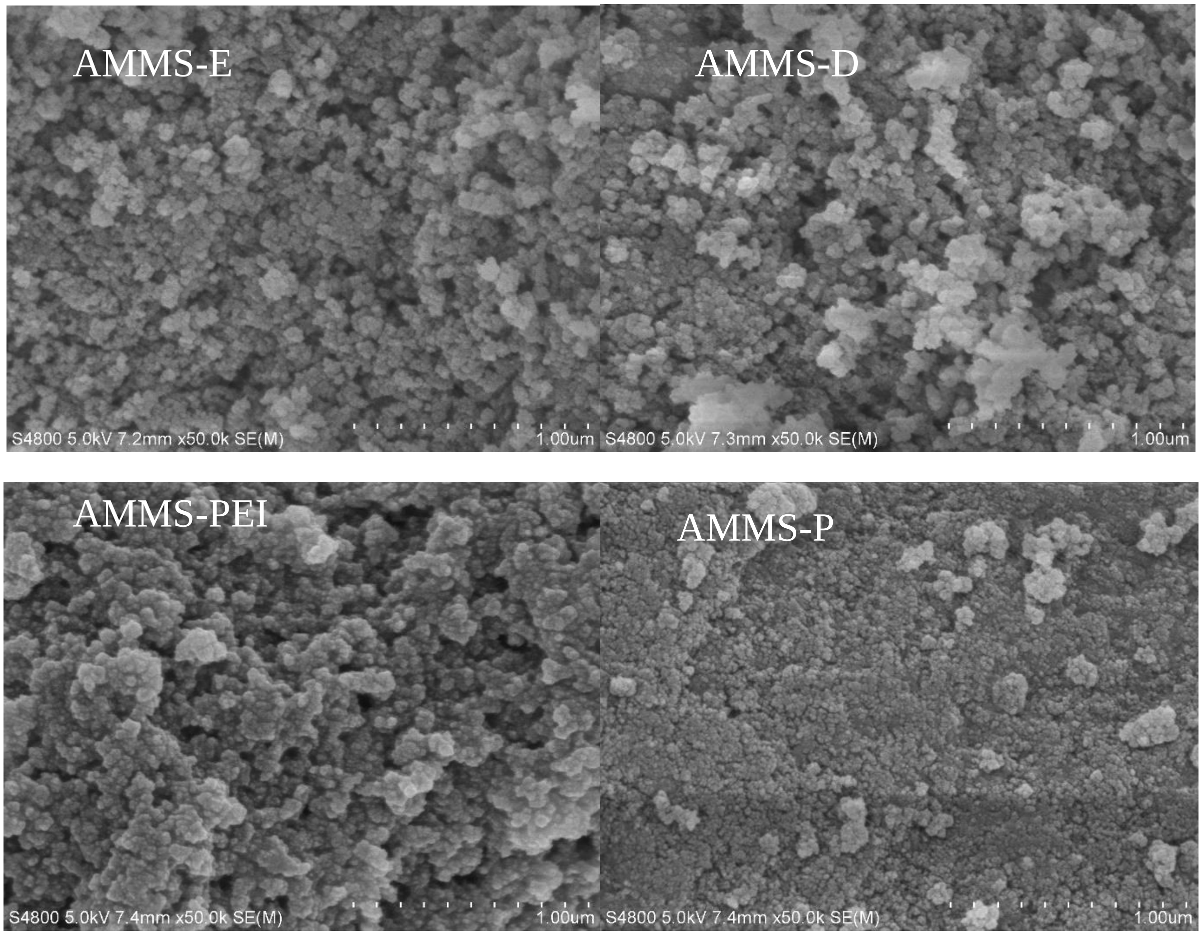

Figure S1 SEM images of AMMS-E, AMMS-D, AMMS-PEI and AMMS-P

Figure S2 N_2 adsorption isotherms of AMMS-E, AMMS-D, AMMS-P and AMMS-PEI

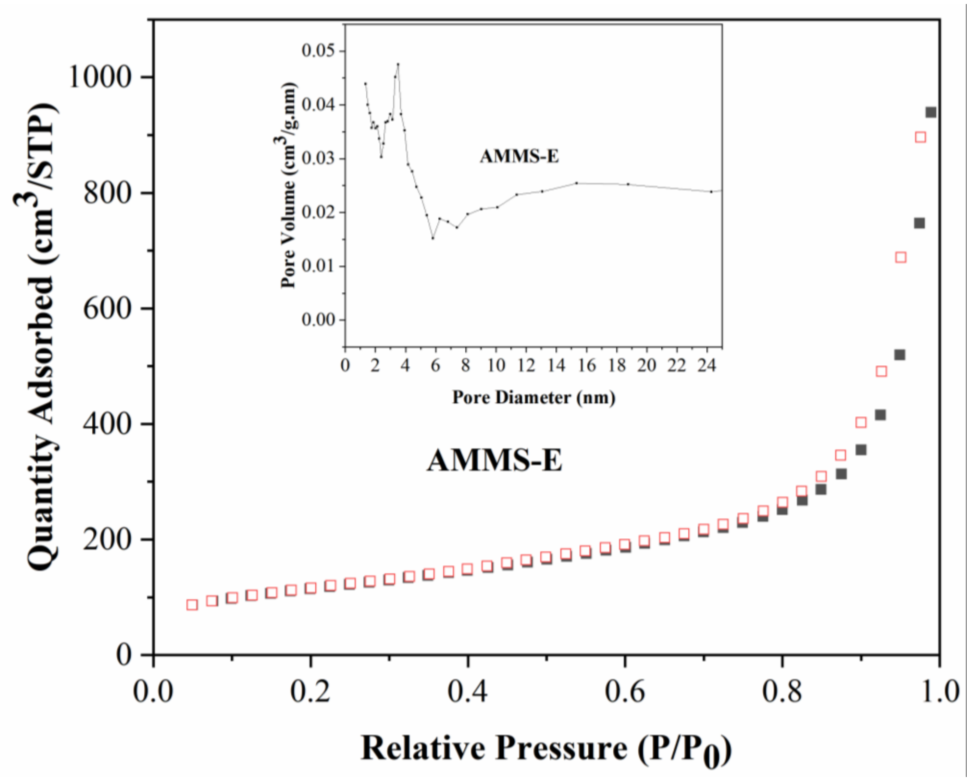

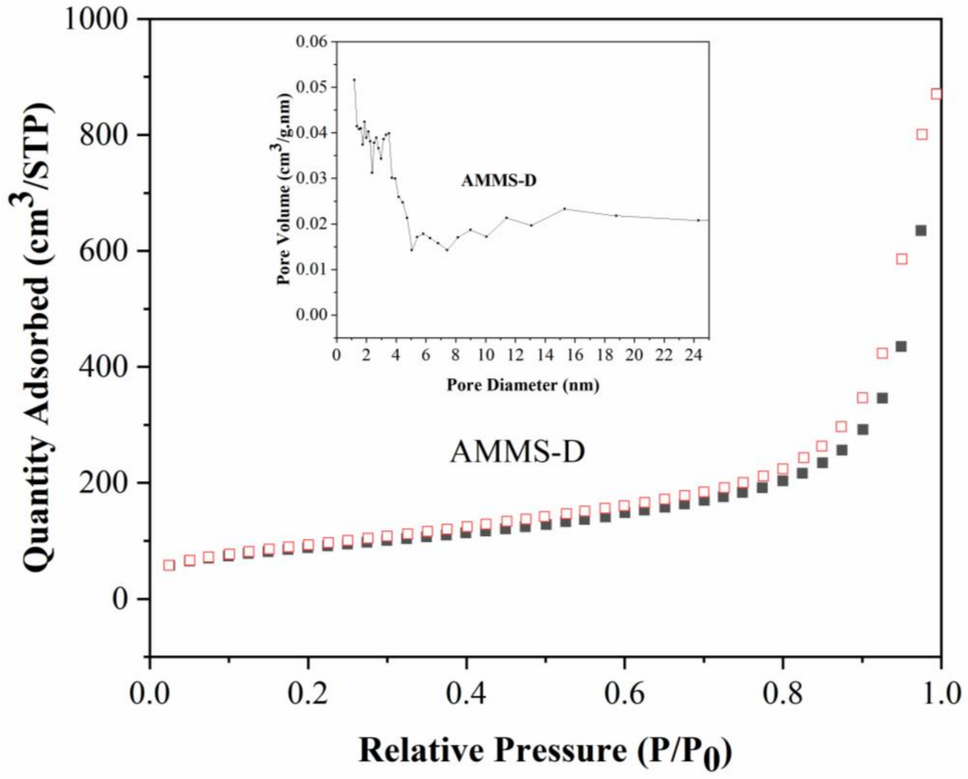

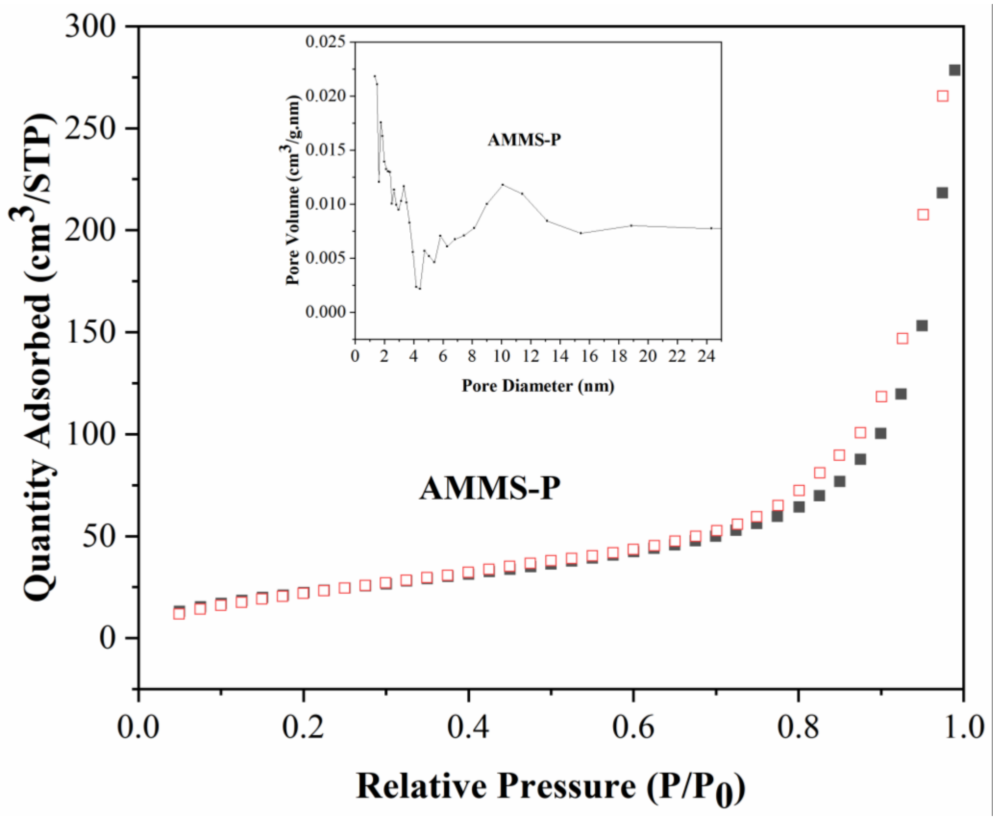

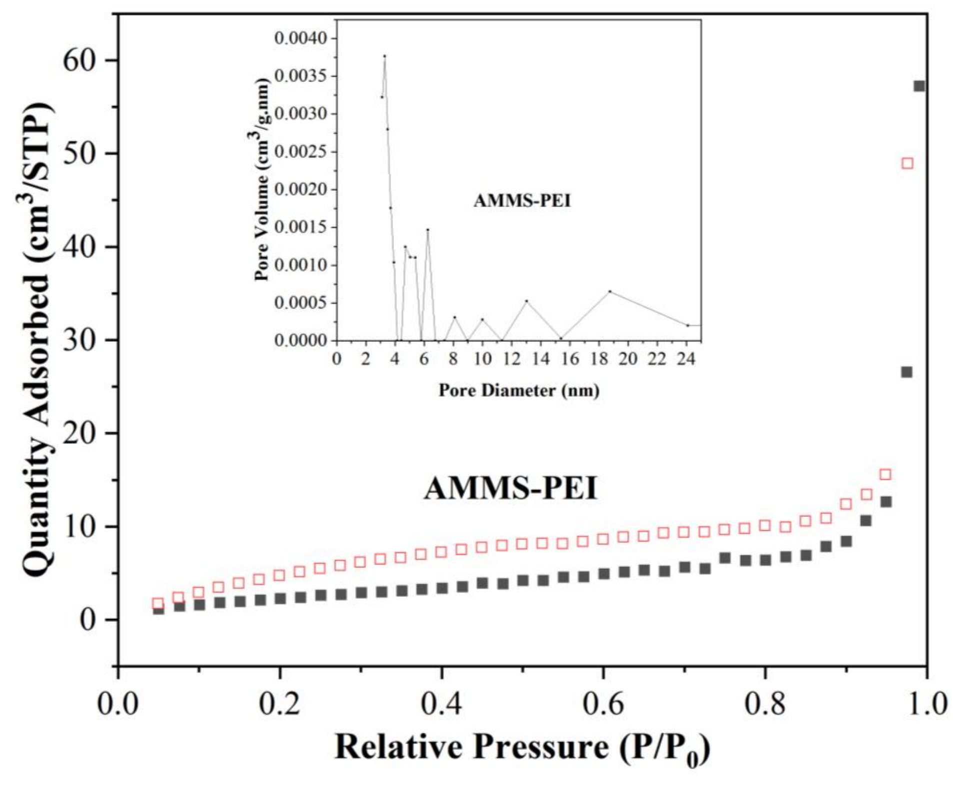

Figure S2. N_2 adsorption isotherms of AMMS-E, AMMS-D, AMMS-P and AMMS-PEI

Figure S3. CO_2 **adsorption curves of MMS, AMMS-E, AMMS-D, AMMS-P and AMMS-PEI**

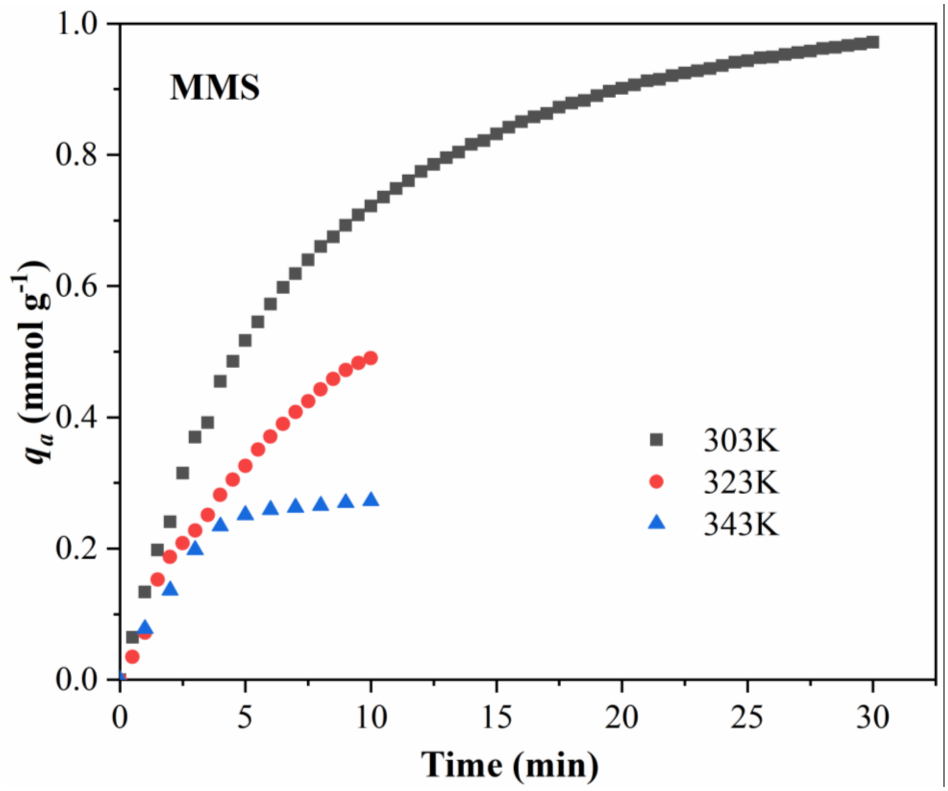

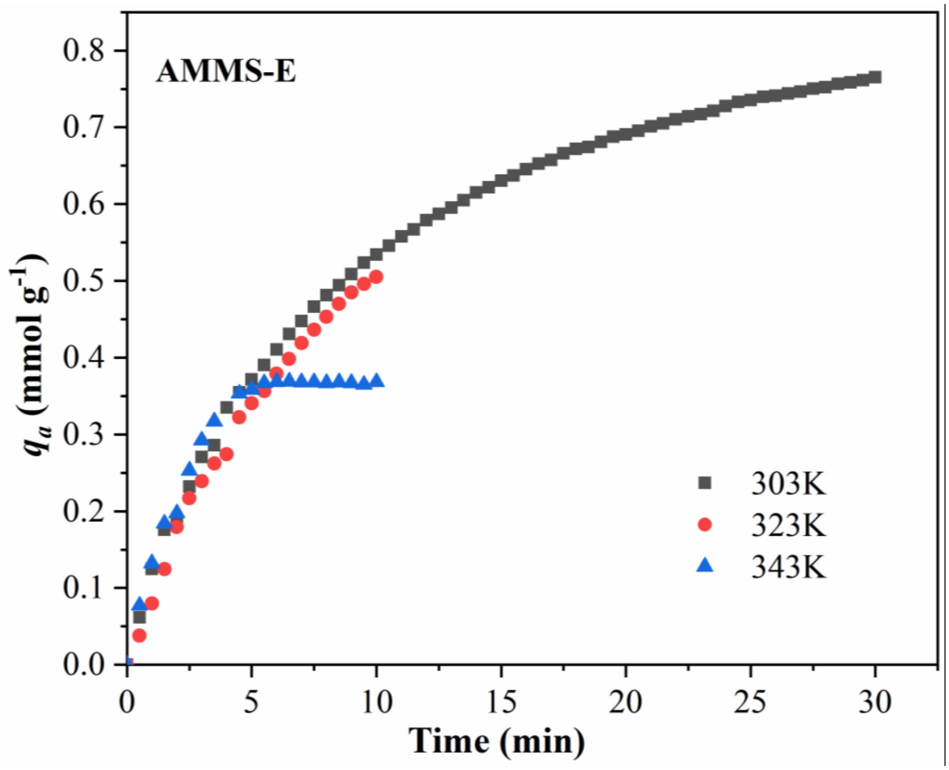

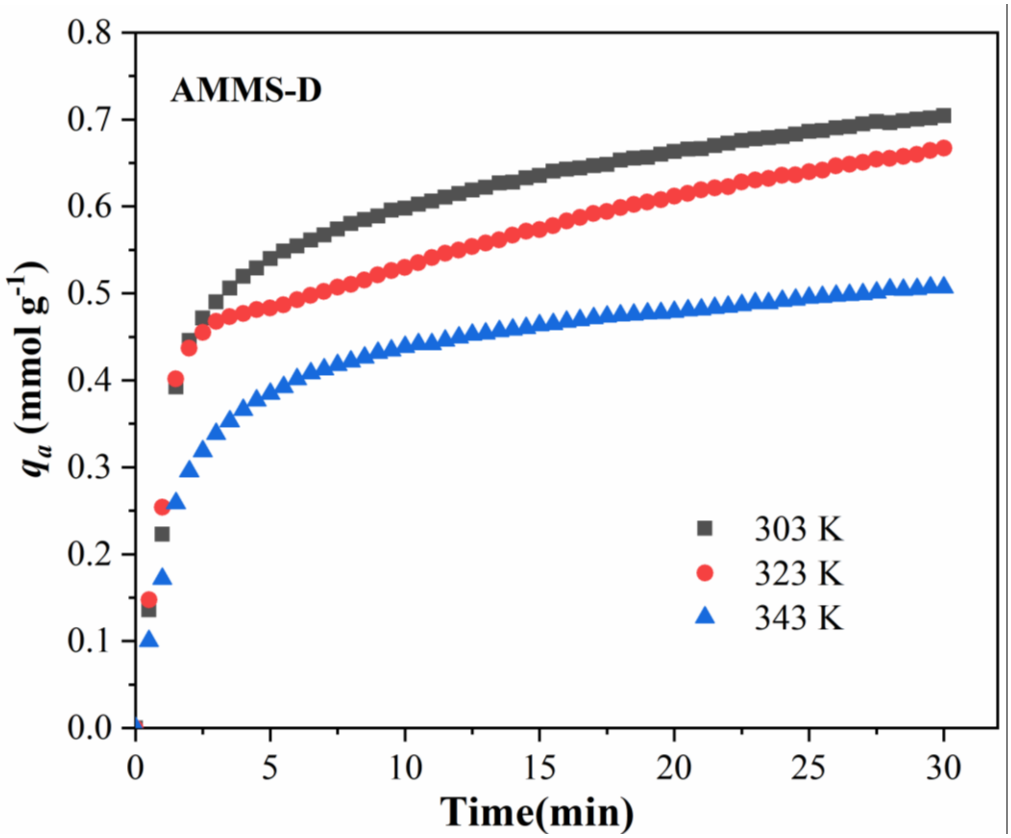
AMMS-D
303 K
323 K
343 K
Time(min)
q_a (mmol g^{-1})
0.0
0.1
0.2
0.3
0.4
0.5
0.6
0.7
0.8
0
5
10
15
20
25
30

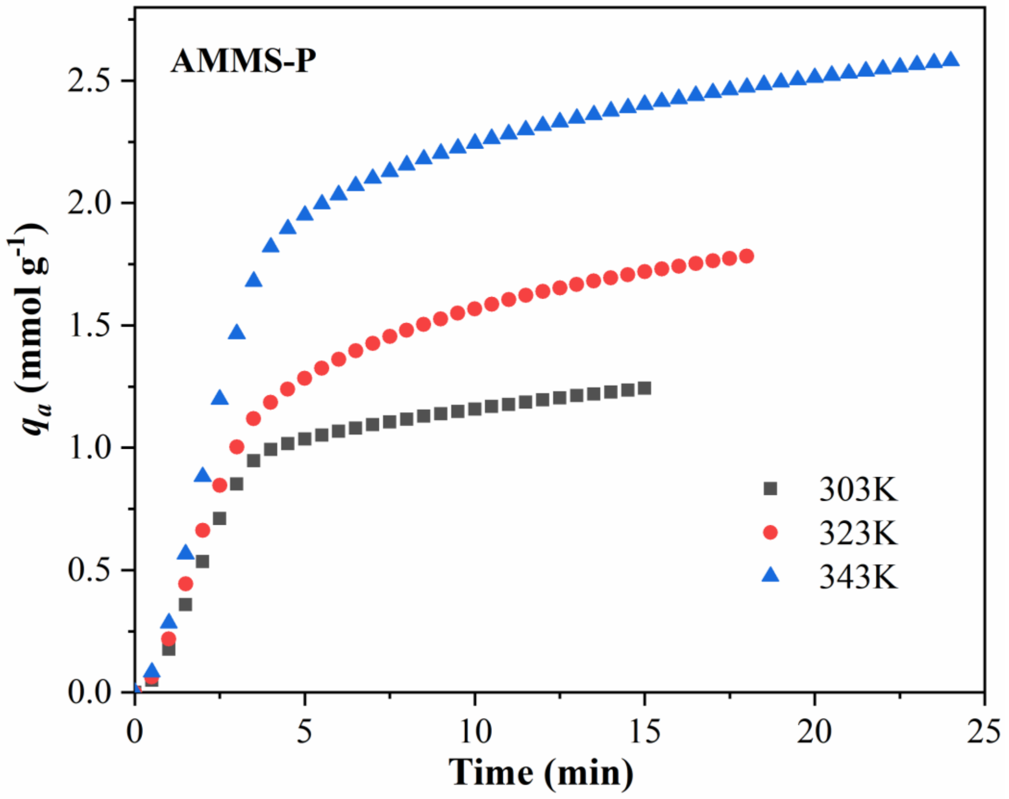
AMMS-P
303K
323K
343K
Time (min)
q_a (mmol g^{-1})
0.0
0.5
1.0
1.5
2.0
2.5
0
5
10
15
20
25

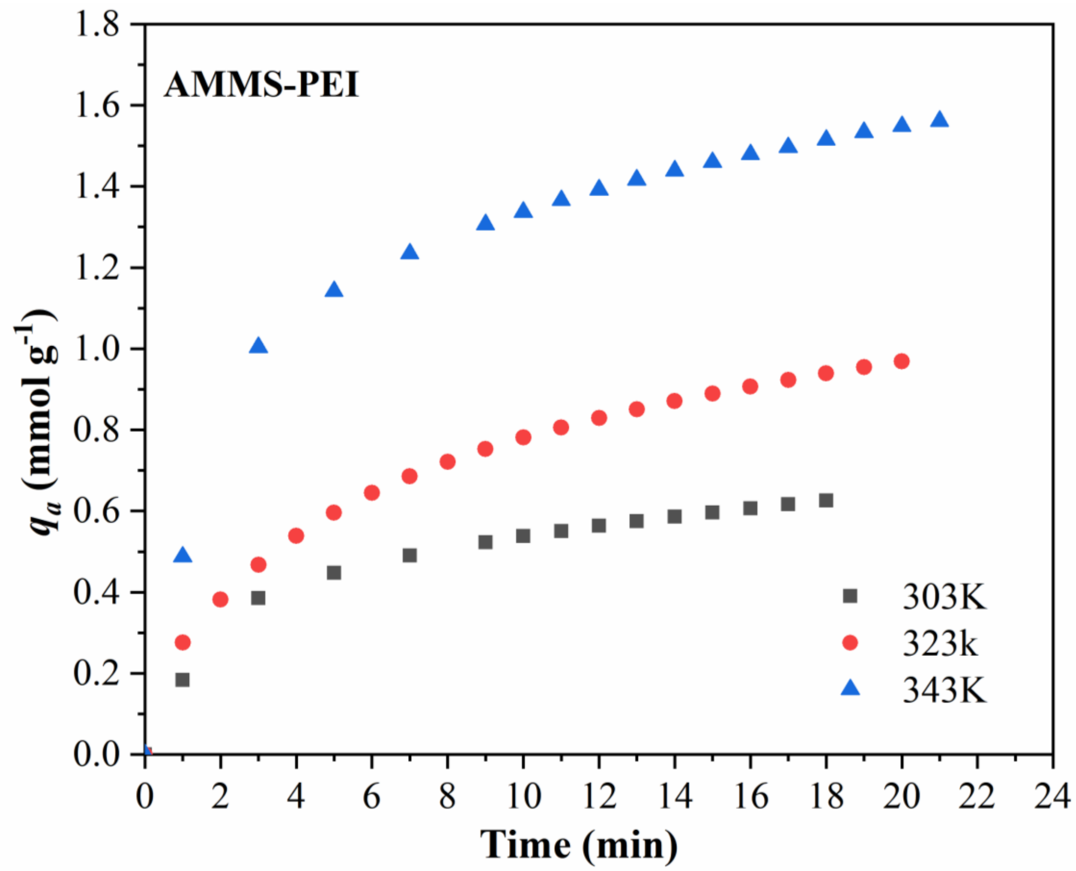

Figure S3 CO_2 adsorption curves of MMS, AMMS-E, AMMS-D, AMMS-P and AMMS-PEI
